# Fourth Annual Meeting of the Ohio State Dental Society

**Published:** 1870-02

**Authors:** 


### Proceedings of Societies.

### FOURTH ANNUAL MEETING OF THE OHIO STATE DENTAL SOCIETY.

[Continued from page 39.]

Dr. Watt read a paper on the use of the mallet in filling teeth; which, however, was not written for the occasion.

Dr. Taft suggested that the members express their views on the subject of the paper.

Dr. Keely announced that there was present a Dentist from Kentucky, and also one from Michigan; and moved that they be invited to participate in the discussions of the meeting.

The motion was agreed to.

Dr. Palmer, on being called upon to express his opinions in regard to the use of the mallet, said the mallet should be a lead one, weighing about eight ounces, with the handle so stiff that there would be no spring to it.

Dr. C. R. Butler had little to say about lead mallets. For three or four weeks he had used a lead mallet a part of the time.

Some twelve years ago, while attending lectures in the old Pennsylvania College, he had an ivory mallet made, which he used from that time up to about four weeks ago. During these twelve years, there have been efforts made, on the part of the profession, in different localities, to construct mallets so as to bring in something that would be acceptable to the patient; and a great deal of genius has been expended in producing machinery for plugging teeth. He had tried a good many of these contrivances, and sometimes against his better judgment, but in order to give them a trial; and he still held that the common mallet is the best formed instrument that he ever tried in packing gold. He had used it, probably, seven-eighths of the time in the last twelve years. He approved the aid of an assistant when he could command the right kind of one, but he had become somewhat familiar with the use of the mallet, so that he could control the whole matter in his own hands. There had been much effort to produce lighter mallets; those that have elastic spring heads, &c., but he had not seen any that were as good as the old kind. The objection had been made to it that it was so heavy; but gentlemen that made this objection and favored lighter ones, have gone so far in the other direction as to make the mallet weigh ten or twelve ounces; but have settled down on the notion that they must have a lead mallet of about eight ounces. He had used one a week weighing ten ounces, but that was too heavy. In his own experience, he had teeth plugged by hand pressure and with different kinds of mallets. He had a tooth plugged some five weeks ago by a mallet that he had used ever since, except a short time; it was lead—perhaps one-fourth tin—the inside of wood, and weighs a little over four ounces. He must say the operation made with it on his own teeth was one of the most comfortable he ever had made; that is, the sensation on the teeth was comfortable, during the operation, and since the operation there has not been the slightest degree of discomfort. He had suffered much more from operations before this, not nearly as large as this. He felt satisfied that the lead mallet, so far, had given better results, packing the gold more solidly, and with less pain to the patient.

Dr. Watt remarked that the principles laid down in the short paper he had read are recognized as the laws that govern matter. If a heavy mallet feels more comfortable to the teeth than a light one, then it was a mistake to condense the gold without pounding the teeth well. For the tooth's own sake, if it needed pounding, he supposed the heavy mallet would be better.

If you would perform the operation of plugging a tooth without jarring it, the momentum must be given by a brisk motion of a light mallet, and not by the slower blows of a heavy one. This principle of natural philosophy had been recognized for four thousand years, and they would either have to change the laws of matter and motion, or else prove that it was desirable to jar the tooth and bruise the periosteum. The month's experiment of the gentleman amounted to nothing, when it contradicted the laws of nature.

He knew of nothing so elastic as ivory, unless it be glass. What we mean by elasticity is, that property which causes matter, when forced out of position, to come back again to its natural position. If it is desired that the *force* of the stroke, in pounding the gold in the teeth, should cease as soon as it is made, an elastic substance of this kind is preferable. You want the force exerted on the plugger, and instantly cease there; and do not want the force diffused to the periosteum and surrounding parts.

Dr. C. R. Butler said he had great respect for Dr. Watt's science and philosophy, but facts are stubborn things. Twelve years experience or more, in the use of the mallet, he thought had taught him something. He believed he had been able to get a power by which he obtained better results than ever before; but because he had an instrument with which he could knock a tooth out of a jaw it was no reason why he should do so. He did not advocate the use of excessively heavy mallets, but of one of moderate weight. He had made operations of the most difficult kind with the mallet, and had less complaint made by patients than when filling by hand force. Even children object less to the mallet than to packing by hand force. He was, perhaps, as strongly opposed to the use of the mallet as any, until he became convinced of its superiority. He had been operating right in a house where another operator used hand force, and he the mallet, and he heard the patients of the other complain a great deal more than his.

Dr. Buffett had been rather skeptical in regard to the heavy mallet; there were many things about it he could not harmonize. He had used the lead mallet but half a day, making four operations with it, and was better pleased with it than he anticipated; was going to give it a further trial. Patients did not complain so much of irritation. There seemed to be a feeling of more solidity than when a light mallet is used.

Dr. Watt had spoke of the force rebounding when a light elastic mallet is used. The speaker inquired if we did not want the force diffused? Thought perhaps it condensed the gold better than a quick blow, the force of which fell at one point.

Dr. H. A. Smith thought the objection to the mallet was that the force was applied in only one direction, and therefore, those that oppose the use of the mallet say that their fillings leak. He had been accustomed to the use of a mallet made of wood, at first, then took hold of the ivory mallet. There was no comparison between them. He used a moderately heavy one—rather too heavy he thought. It was the most elastic substance he had ever tried. He gave it preference over lead.

Dr. G. W. Keely remarked that he had been using the mallet ever since it was first proposed; had generally used an ivory one; the weight probably not more than four ounces. For the last few weeks he had been using a lead mallet; most of his patients liked it better; the dead sound of it seemed more pleasant than the harsh sound of the lighter mallets.

Dr. Watt thought much of the difficulty with the lighter mallets was the kind of persons employed to use it. If awkward malleters are employed, you must have slow strokes, and, as the velocity is slight, a heavy mallet must be used.

Dr. G. W. Dunn.—A few weeks since was condensing the filling of a tooth, when an automatic mallet was brought into the office. He used it in finishing the filling. It worked with a great deal of velocity. The way the gold gave way under it showed that there was a great deal of force. To his surprise, the patient experienced less unpleasant sensations than in the use of a mallet. He had tested it on a tooth of his own, and he was convinced that the blow coming with greater velocity was less painful than he expected; and he thought the experience he had was common with the principle stated by Dr. Watt. The greater velocity, he believed, likewise condensed the gold better.

Dr. Palmer said if it was not for the great respect he had for Dr. Watt, he would like to make some remarks on this point. This thing of filling teeth with the mallet is very peculiar. He had been experimenting in that way since the idea was first advanced, or nearly so. He had used three or four in succession, one after the other. First, a light one with a tolerably long handle. He had used it for some time, and supposed it was about the right thing. Dr. Atkinson, on seeing it, told him he was fooling away his time in using it. He then got one some heavier, and found, after a careful trial, that the increased weight was an advantage. He had finally got to using the lead mallet, and believed it still better than anything he had used, and believed better results could be obtained with it. He would not undertake to go into the principles of philosophy involved in it, but he was convinced of its excellence from his experience in its use, and comfortable sensation experienced by patients compared with the use of the lighter mallet. The gold is as much more perfectly packed by the lead mallet, than by the ordinary mallet, as that packed by the ordinary mallet exceeds that done by hand pressure.

Dr. Butler.—I would ask if you do not use an assistant constantly?

Dr. Palmer.—I have for the last two or three years.

Dr. Watt, after speaking more at length upon the philosophy of the principle he had advocated, remarked that he did not put much confidence in what patients might say in reference to the absence of pain in operations on the teeth. About twenty-six hundred patients to whom he had applied the galvanic battery, when conducting experiments in extracting teeth for them, had testified that it did not pain them at all. He thought the testimony of about one thousand of them could not be relied on. He had given persons bread pills, in cases of extreme pain, and they have affirmed that they were immediately relieved; when he saw from their very countenances that they were suffering the greatest agony. The principles he had set forth were the principles that governed matter and force, and for four thousand years have been recognized; and the experiences of a few cases a month were not sufficient to establish anything to the contrary.

Dr. Spellman.—Had no experience with the lead mallet. He did not, however, believe that Dr. Watt's theory with regard to matter and motion was correct. He theorized at some length on the subject. He thought with the elastic mallet a vibrating stroke was given, which caused the uncomfortable sensation complained of by patients. He thought that the reaction that takes place in the particles of an elastic mallet reacts upon the instrument, and we do not get that same kind of a telling blow as with a mallet that has not that elasticity. It is a well established principle of philosophy that action and re-action are equal.

After some further remarks on the subject, by Drs. Watt and Spellman, the meeting adjourned until 9 A. M., the following day.

SECOND DAY.

The committee appointed to consider the propriety of changing the certificate fee charged by our State Board of Examiners, reported against making any change.

*In Reference to this Subject*—Dr. Watt remarked, in reference to the opposition made last year against the fee charged applicants, by the Board of Examiners, that he thought the opposition had died down. Still we must be wide awake, and ready to ward off any opposition that may be raised against the measure. A part of the opposition, at least, originated with a constitutionally discontented man—a confirmed dyspeptic—who had been hit on the back with a sour apple. He had been given a sweet apple now, and had moved away, and would have nothing to do with the Legislature this year.

Dr. Taft said: I think if this report is adopted, as it should be, every member of the association and profession, who is in sympathy with the law, and especially those who have passed this examination, will be ready at all times, and whenever any opposition is manifested, to come to the rescue, and do whatever they can to squarely and promptly meet it. It is possible there will be no opposition this winter; but we can not tell what may come. The very man to whom we are indebted for the passage of the law at the outset, said, if it be true that a fee of twenty-five dollars is charged for these certificates, he would go for the repeal of the law. But he has got right on the question. There were others that could only be quieted by the promise that at this meeting the Society would consider the matter, and do what seemed best. Of course the active friends of this law are increasing all the while. Those who pass this examination, and receive the advantage accruing from it, ought to be desirous that everybody be brought on the same footing with themselves. We should endeavor to bring our profession up to a common level. The argument used, is that we are trying to crush others. It is not true. It is the duty of every member to help all worthy members of the profession, and bring them up to a high position.

Dr. G. W. Keely said he was decidedly in favor of the adoption of the report. The duties of the Examining Committee are attended with a good deal of expense; and the Society should remember that they are responsible to this committee for expenses in coming to Columbus to attend the meeting of the Board. Who should bear this expense, if not the men who get the benefit of it? He did not see how they could afford to cheapen it. It was cheap enough at twenty-five dollars. The opposition to the law, he believed, would all die out.

Dr. DeCamp—Was very well satisfied that, if the difficulties in regard to the matter were removed, there would be other points complained of. He made the motion in order to bring the subject before the Society. A gentleman had complained to him, last summer, about all applicants having to come to Columbus to be examined. He thought the price low enough, but he objected to the requirement for every one to be examined at Columbus. There were ten in his neighborhood that desired to be examined. This gentleman did not see how he could take time to leave his office to go to Columbus for examination. If the examination fee is reduced, of course the fund arising from this source will not pay the expenses of the Examining Board.

Dr. Blount, of Springfield, thought there was no doubt but that all the practicing physicians, or the greater part of them, would use their influence to maintain this law. He had conversed with several in his place, and they all said they would do all in their power to sustain it, as they have one similar to it.

Dr. Watt thought the medical law quite different from that regulating the practice of Dentistry. Physicians generally think they are nearly alike; but when the difference is pointed out, they are perfectly plain. In their law there is no restriction in regard to forming societies. Suppose there is a rutebago doctor in one corner of Franklin County, and an iron doctor in another corner, and two or three quacks in other parts of the county; are they going to stop practicing? Not at all; they simply form a Franklin County medical society, and issue their certificate to one another. But when you tell these men that according to law certificates can be granted only by the State Society and its auxiliaries, and that we have only the State

Society, and our constitution provides for no other, they see the difference.

Dr. C. R. Butler said there was still another thing in regard to this law that might be noticed. There are those who complain of the expenses of examination, but when you come to take into consideration this expense, compared to that required to come through the acknowledged doors into the profession, it is very small. There are men who have been practicing medicine for years, and when they find they must pass the examination, and receive a certificate to allow them to practice, they prefer going through a regular course than do this. There are medical students in Cleveland who, rather than go before the Board and be examined in this way, went and took a regular course in the schools. To his mind, that was a noble and manly course.

This is the acknowledged door through which all professional men come, and no other should be acknowledged. It would be folly for him to set up a claim to theology or law when he had never taken any steps to put himself in a position to entitle him to that claim. There are men that do not like any restrictions ; but the law is not made for the law abiding, but for those who will not be governed by law if they can help it. There would be opposition to the law if the fee were reduced to $10. We have the best law, in regard to our profession, in any of the States, and he thought we would get it still better after a while, instead of having it repealed. Some persons practicing Dentistry in the vicinity of Cleveland, say they would be examined, but can not get time; can not leave their business. He thought their patients would think more of them and give them enough extra to pay their expenses, and would honor them for such a course. No law is good for anything unless executed. The executors of the law are those who recognize its validity and hold it up. This law simply requires a co-operation on the part of those who would like to be recognized as professional men. It needs only that they put their hands and hearts into the work, and hold up the standard and never let it trail.

Dr. Decamp said: In his section of the country there was some talk about opposition to the law, by the establishment of Dental colleges in different parts of the country. A man there was getting ready to establish a Dental college, and says there are two men now ready to go into it and take professorships. The design was to allow all within certain limits to attend the college until they had obtained certificates, and then let the college go down.

Dr. Herriott apprehended no trouble from that source. They would find considerable expense and trouble in establishing such colleges. In his county, and the two adjoining counties, the law is carried out, and several who undertook to practice Dentistry have not been able, and quit.

The report was then adopted.

On motion of Dr. Rehwinkle, the second topic, "Diseased Antrum, and its Treatment," was postponed until the next annual meeting.

*Mechanical Dentistry.*—The subject of Mechanical Dentistry was then taken up.

Dr. Herriott read the report of the Committee in reference to vulcanized rubber. (The report condemned the use of rubber plates, on account of their injurious effects.)

Dr. Rehwinkle was in favor of the principal part of the report; but could not subscribe to all that was set forth therein. By endorsing the report in full, we simply use the State Society, through its moral force, to condemn and assert things which are not proved true. The report asserts that the use of the rubber plate produces disease. He was not able to say that this is a fact. While he would not recommend any one thing exclusively, neither would he condemn one, when he is not convinced, beyond all doubt, that it is injurious, as claimed to be. He had never seen a single case of ptyalism produced by rubber. It might be aggravated by it; and this might result from other things. They must be eclectic in their practice. One case might require a particular kind of plate, and another, a different kind; he could not consent that the Society either endorse or condemn any particular thing.

Dr. Herriott thought the reasons given in the preamble sufficient to show that the resolution should be adopted. He had in his mind two cases of ptyalism produced from the use of the rubber plate. It may, perhaps, be produced, also, by other causes; but he was sufficiently satisfied of its injurious effects to say that its use should be condemned.

Dr. Way knew that an objection had been made to rubber on account of its being a non-conductor; but the same objection could be made to several other kinds of plate. He didn't see why they should particularly anathematize rubber. If the resolution proposed was merely discriminating, he would endorse it.

Dr. Rehwinkle understood that the State Society would better avoid any endorsement or condemnation. They could only hold fast the general principles that must govern the practice of their profession, leaving each member to use his own judgment as to what was the best to be done in particular cases. He, therefore, moved that the resolution be laid on the table.

A vote being taken, the resolution was laid on the table.

Dr. Wright, of Louisville, by invitation, spoke upon the subject of Rose-pearl plate. Fifteen months since he commenced the use of rose-pearl plate, and had been using it ever since. Like every other kind of work, it has its advantages and disadvantages. He didn't know any kind of plate that didn't have some disadvantages; but for a cleanly, good and pleasant plate he knew it could be well recommended. It required no more skill to make them than to make rubber plates. The material is in two forms—the hard sheets or plates, and in the granular form, for building on the gums. The plates can be softened or moulded into a continuous gum-work. Metal plates can be used if desired with rose-pearl attachment. There is an objection to it on the part of some, from the time required to make it, but he thinks this a slight objection. It requires some sixty or seventy hours to finish it, but the work to the Dentist is not much more than to make a set of teeth on rubber or other material.

Dr. Herriott inquired if the material shrank so as to sometimes crush the teeth, as formerly.

Dr. Wright said he never saw any teeth crushed by the use of it. As to its shrinking or warping, it is all a mistake. The first work made, two years ago, might have done so, but if it did, the difficulty had certainly been obviated.

Dr. Herriott—Can it be easily repaired?

Dr. Wright—As easily as any other work. Some claim that it is more easily repaired.

Dr. Smith—How long would it require to finish an attachment of other material on the base of rose-pearl?

Dr. Wright—That would depend upon the rapidity of the manipulator. It would take some a couple of hours; some, perhaps, four hours. That kind is only used for cheap work, gotten up, I believe, to attach to plates of rubber.

Dr. Buffett—Which do you regard best.

Dr. Wright—The rose-pearl attachment is best. I prefer to attach each tooth by tin or silver, or any way you may choose to the plate, then it is all covered up with rose-pearl material.

An intermission of ten minutes was then given, to allow members to examine specimens of the rose-pearl work.

Dr. G. W. Dunn made some remarks in reference to porcelain work. He said his convictions were stronger than ever, from his continued experience with it, that it has more demands upon the profession, and upon the public, than perhaps they are aware of. The results, from its being worn by patients, had been very satisfactory. A species of work might be objectionable to the patient and still it might not be apparent. Especially was this the case with rubber. Even when it had produced bad effects in the mouth, patients expressed themselves as being pleased with it. As remarked by Dr. Wright, there were no kinds of plate that had not some objections. We should seek for that which will be best, and make the most perfect denture. He supposed it was known to all that the mineral plate, as presented in a continuous solid piece, is impervious to the fluids of the mouth, and not open to the objection that all styles of work are, that have a combination of materials entering into them. He was aware that the objection is urged, perhaps not with any design to disparage the work, that it is not strong, and can not be easily repaired; but, by experience and a little care, it can be well mended—perhaps as easily as most kinds of work in use. He thinks a great detriment to the mineral plate, as well as some others, had come from the fact that the cases which don't give satisfaction are those that more especially come to our notice.

Dr. Rehwinkle remarked that in 1867 he had some porcelain teeth put in by Dr. Dunn, but that he had a short time previously had some teeth extracted, and that his mouth was undergoing changes at the time, and it was impossible that as accurate a fit and adaptation could be made as under other circumstances; still he could wear these porcelain teeth very well now, although a plate made from an impression taken recently would do much better. Under the circumstances, he thought the set of porcelain teeth worn by him was no test as to the merits of this material.

Dr. W. E. Dunn said he must repeat what he had often said before—the longer he used mineral plate, the more strongly he was convinced that it was capable of being used to advantage by the profession, and that it is a plate that will give satisfaction, when properly made. Many persons who had worn various other styles of work, and then had worn the porcelain, were better satisfied with it than any kind they had ever worn. He thought it was a duty we owed to the public that the importance of this style of plate be made more fully known.

Dr. Keely said he understood that it was almost impossible to get as accurate a fit with this style as with metal or rubber; but perhaps the fact that the tissues of the mouth are not so injured by it, is because they do not fit so closely as to disturb the tissues of the mouth.

Dr. Dunn thought he could demonstrate that the mineral plate worn by his patients fit as accurately, or approach as nearly to accuracy, as any kind that were worn; although many of the profession had a different impression in regard to this matter. In almost all cases, the adaptation of the plate to the mouth could be made almost perfect.

Dr. Herriott had two cases where porcelain was being worn by patients who had worn gold and rubber before, and were better pleased with the porcelain.

Dr. J. Williams gave some reasons why he regarded porcelain plate as less productive of disease in the mouth. One was, that it is a good conductor of heat, and allows heat to pass off readily from the tissues beneath it. Another is, that it is more easily kept clean than anything he had ever seen worn in the mouth. He thought as good fits could be obtained with porcelain as any other style of work. He had the pleasure of saying that so far as beauty of appearance, cleanliness, durability and healthfulness are concerned, he knew of nothing that can surpass or equal the porcelain plates. An experience of three years warranted him in saying that it gave his patients better satisfaction than any work he had ever made.

Dr. DeCamp remarked that the State Dental Association, two years ago, endorsed this mineral base and extolled it very highly. That endorsement went all over the country; and yet there are but comparatively few making that kind of work. In making gold plates an absolutely perfect fit can not be obtained. In fitting the plate there would always be a little variation. That was, perhaps, one reason why we did not see that irritated appearance of the mouth mentioned in the discussion yesterday. We all know that gold is a good conductor; yet the freeness of the mouth from this appearance is most probably because there is not a perfect fit. If we want to give our patients "fits," we generally resort to rubber. [Laughter.] We can approximate more nearly to the perfect fit with rubber than any material we know of; and the probabilities are that it is not the material in the rubber, not the mercury, that produces that redness and soreness of the mouth, but an exclusion of the atmosphere from the mouth. With the porcelain plate we know there can not be such a perfect fit obtained, and there is no evidence of diseased mouth on this account where it is worn. He presumed the porcelain plate could not be ground to fit so perfectly, but that more air would get under it than under the rubber plate. That was probably the reason why there is nothing of that red appearance of the mouth where it is worn. If we would take the number of persons wearing gold, the number wearing rubber, and the number wearing porcelain, we would probably find a large proportion of persons wearing rubber, better satisfied with it than with any other material. Continuous gum-work approaches more nearly a perfect fit than porcelain, and is really the finest kind of work made; is serviceable, and the material is a conductor. If it could be generally adopted, he thought it would give more general satisfaction than any kind of work we could adopt.

Dr. Spellman admitted that the remark of Dr. DeCamp was probably true, that a greater proportion of those wearing ar'ificial dentures give their preference to the rubber. But there are more wearing rubber than any other; the people have a mania at this time for wearing rubber, and they fall into the praise of it from the general custom, rather than from their judgment concerning the value of the material. The judgment of patients usually, in regard to any style of work, is worth nothing. In many cases we see diseased mouths of which the patients did not complain; not knowing, indeed, that their mouths were diseased. He had seen persons return and say they could not manage their plates; that they did not fit; when the plates did fit perfectly. On the contrary, others have declared the plates fit perfectly when there was no fit at all. So the testimony of patients amounts to nothing.

Dr. W. E. Dunn believed that the mineral or porcelain plate is capable of being adapted more perfectly to the mouth than continuous gum-work. He had made continuous gum-work, put it into the mouth; but, from his knowledge of the two materials, he thought the porcelain preferable.

Dr. Rehwinkle believed he could satisfy any worker that the continuous gum-work was more acceptable and comfortable than the porcelain could be.

Dr. G. W. Dunn desired to refer to a comparison in regard to the cleanliness of the different styles of work. Cleanliness was certainly an important point to be attained in securing a denture that would be satisfactory to the public. We secure that better by any material which is continuous or of one material, for the whole structure. The interstices that occur in most kinds of work become filled with extraneous matter, so as to become offensive and deleterious to health.

Dr. Rehwinkle thought there were many advantages in the the porcelain work. This was true of all the different styles of work; but the varieties of cases they were called upon to meet were such that they could not find anything to meet all cases. If everything were equal, in porcelain we would have such a material as would leave nothing to be desired. While it is true porcelain will not take the nicotine of tobacco, for instance, which silver or gold plate will, yet the surface of the plate which goes next to the gum will tak as much of the food as rubber or gold plate.

Dr. Watt said that he had seen a good many sets of porcelain teeth, but that all had one radical objection—that is, they were thick, bulky and clumsy. This was the case with the best specimens he had ever seen. He thought the necessary strength could not be obtained without making them too clumsy. The first time he ever saw his friend, Dr. Dunn, was in one of our conventions, at Cleveland. The Doctor did not know as much about porcelain at that time as now; but he was investigating earnestly, and he felt convinced then that whatever there was in this material he would work out, and he confessed he never saw as fine specimens of work of that kind as Dr. Dunn had made.

The meeting then adjourned, until 3 o'clock P. M.

*Afternoon Session.*

The meeting re-assembled at 3 o'clock P. M. The President appointed as a committee to nominate officers Drs. Blount, Jennings and DeCamp.

After some time spent in miscellaneous matters, the committee on Nomination of Officers reported the following names: For President, Drs. H. A. Smith, F. A. Rehwinkle; First Vice President, Drs. L. Buffett and B. T. Spellman; Second Vice President, Drs. N. W. Williams and C. R. Taft; Recording Secretary, C. R. Butler and W. M. Herriott; Corresponding Secretary, Drs. I. W. Williams, A. W. Maxwell; Treasurer, Drs. Geo. Watt and A. Berry.

The members proceeded to ballot, and the following officers were elected:

For President—Dr. F. A. Rehwinkle, of Chillicothe.

Vice President—Dr. T. Buffett, of Cleveland.

Second Vice President—Dr. N. W. Williams, of Xenia.

Corresponding Secretary—A. W. Maxwell, of Galion.

Recording Secretary—Dr. W. M. Herriott, of Zanesville.

Treasurer—Dr. George Watt, of Cincinnati.

The remainder of the session was devoted to miscellaneous business, when the Society adjourned until 7½ P. M.

*Evening Session.*

Dr. Horten, in retiring from office, made the following remarks:

"*Gentlemen of the Ohio Dental Association:*

"In presiding over you, the past year, there have been a great many things that to me have been very pleasant, the memory of which will not leave me very soon. I hope you will manifest towards my successor and successors in office, who may preside over you, the same consideration that you have towards me; and I have no doubt my successor will leave the Chair in as happy a state of mind as I do. I thank you, gentlemen, heartily for the courtesy you have uniformly manifested towards me."

Drs. DeCamp and H. A. Smith were appointed to conduct the President elect to the Chair. On reaching the platform, Dr. DeCamp said: "Gentlemen, I have the pleasure of presenting to you Dr. Rehwinkle, whom you have been pleased to elect as your President for the ensuing year. Dr. Rehwinkle was greeted with hearty applause, and addressed the Society as follows:

"*Gentlemen of the Ohio State Dental Association:*

"I thank you for the honor you have been pleased to bestow upon me. I assure you I appreciate it as highly as any honor ever bestowed upon me. Yet it is with a great deal of misgiving in my own ability that I accept the position to which you have chosen me; and I make a personal appeal to each and every one of you to bear with me in striving to fill an office to which I am unaccustomed. I give you my assurance that so far as my individual efforts are concerned, I shall never lack in trying to do what I can to advance the objects and interests of the Society, and cultivate a friendly spirit amongst the members, and to oppose anything that would do injury to the profession or Society, and to uphold everything that has a tendency to elevate the Dental profession in our own estimation and in the estimation of our fellow men. With these resolutions, I

hope the ability may come as the necessity may require. I can not close without referring to the old saying, that persons never know the value of a good thing till they have lost it; and I know you had a good President in Dr. Horten, and think you missed it in not electing him again. [Laughter.]

### *Evening Session.*

Dr. C. M. Kelsey referred to a case of fracture of the jaw, a description of which he gave at the last annual meeting. The Secretary read a communication from the patient upon whom the operation was performed, speaking in terms of commendation of the success of the operation.

*Standing Committees.*—The following standing committees were elected:

Executive Committee--Drs. W. P. Horton, M. DeCamp and A. A. Blount.

On Membership—Drs. W. M. Herriott, D. R. Jennings and L. Buffett.

On Dental Ethics—Drs. G. W. Keely, C. R. Butler and B. T. Spellman.

On Publication—Drs. J. Taft, A. Berry and Geo. Watt.

Auditing—Drs. H. A. Smith, Geo. W. Keely and C. R. Taft.

Essayists—Drs. A. A. Blount, Geo. Watt, C. R. Taft, D. R. Jennings, W. M. Herriott and C. R. Butler.

Dr. J. Taft read the report of the Rubber Committee. Dr. Horton made a verbal report in reference to the work done by the Committee, &c.

Dr. Kelsey, of Mount Vernon, explained the operation of a new vulcanizer, of which he is the inventor. The models and the explanations given created much interest among the members.

The fourth topic, "Necrosis of the Teeth and Treatment," was taken up and discussed at length.

Dr. Taft—The word necrosis, means death of a bone; but by the term as given, I suppose is understood the death of the tooth, the death of its pulp, and the hard portion of the tooth, so far as it becomes devitalized by the death of the pulp. When the pulp of the tooth is destroyed, the vitality of the bony portion is only partially destroyed; but the dentine of the crown very soon after loses its entire vitality, though there may be some slight exceptions to this. The dentine of the crown of the tooth, and the enamel, are both dependent upon the pulp for their vitality and nourishment. We infer this from the fact that dentine, in the crown of the tooth, loses its sensitiveness very soon after the pulp is destroyed.

However sensitive the dentine may be previous to this time, in the cavity of a decaying tooth, or wherever the dentine is exposed, in the great majority of cases immediately after, and in all cases very soon after the destruction of the pulp, that sensitiveness is all removed; but it is not so with the dentine of the root, that is, it does not wholly lose its vitality for a long time after the pulp is destroyed. The dentine of the root loses in part its vitality, and loses more and more, until by and by its vitality is, perhaps, all gone.

The vitality of the teeth is destroyed by various causes. A very common cause is decay; but death may occur before exposure of the pulp; or it may be destroyed when there is no decay, by blows, by excessive variations in temperature, by undue pressure upon the teeth, by the lessening of the vital forces. Sometimes the teeth are devitalized when they are not decayed, in consequence of a low grade of vitality, induced by disease either general or local. Again, by operations performed at improper times, or when the teeth are in the improper condition for an operation. This matter of necrosis of the teeth is an important one, and we should remember that in many cases it is easily brought about, by influences we are too liable to overlook. When a tooth is devitalized it is not always lost. Nature has so provided that when a tooth is devitalized it may still be rendered quite serviceable, for years; yet a dead tooth is not so good as a living one. This matter of necrosis touches several other things. The enamel is nourished and in proper condition only while the tooth is alive, and the same is true in regard to the dentine. In regard to the treatment, it seems rather paradoxical that we should be called upon to treat a dead tissue. The tooth is not totally devitalized at once, and we have a little vitality to go upon. The periosteum very frequently, and from very slight causes, becomes irritable, and the tooth becomes sore, tender and very sensitive, and must be managed very carefully if we would carry it along in good condition. Especial care should be taken in keeping the teeth clean. Nature designed them for use. If not used, they suffer. An idle tooth is far more likely to be diseased than one that is made to perform its proper functions. So these teeth should be properly used for mastication. It is very certain, if not used, foreign substances will lodge on them, hastening decay and disease.

In decay, the dentine usually is devitalized in advance of the decomposition. But it is not so in all cases. The vitality only yields its position when it is forced back; and its hold is much stronger in some cases than in others, so strong that it only gives way when the house it lives in is torn down around it. My impression is, that vitality will remain in certain cases, even until the dentine is partially decomposed.

The vitality, of course, depends upon two things. First, on the vital force or the ability it has to maintain its position; and upon the character of the fortification in which it is entrenched. If the tooth structure is of good character, and perfect, it will maintain its position right up to the line of battle all the time. In that case, it calls out to the Dentist for help; and with care and good work we are almost certain that what we do will be received kindly by nature and be effectual. But if the vitality leaves its domain, and runs away upon the approach of the enemy, there is far more difficulty in the treatment.

Dr. Watt thought Dr. Taft had made an omission in leaving out of his remarks any reference to the elements, or kind of enemies that make the attack and destroy the vital forces. He said he had been able to find four varieties of dental decay. Three of them are the well defined varieties generally given; then another called chemical abrasion. He then spoke, at some length, on the effects of acids upon the teeth.

At the close of this discussion, Dr. Palmer made some additional remarks upon the subject of mallets.

On motion of Dr. J. Taft, the Chair appointed a committee to select delegates to the next American Dental Association. The Chair appointed Drs. J. Taft and C. R. Butler said committee.

Dr. Butler made some remarks in reference to pluggers, exhibiting models.

Dr. Watt, also, followed in a few remarks on the same subject.

On Motion of Dr. Horton, a vote of thanks was tendered to Rev. G. W. Phillips for his services in opening the sessions of the meeting with prayer.

The Committee on Arrangements were recommended to consider the propriety of continuing the next annual meeting three days. Dr. Horton and others spoke in favor of the proposition.

The following persons were appointed delegates to the American Dental Association, to meet at Nashville on the first Tuesday of August next: Drs. E. C. Sloane, C. R. Taft, W. P. Hall, N. W. Williams, D. R. Jennings, E. J. Way and C. Bradley.

On motion, Drs. Taft and Butler were appointed a special committee to see that delegates attend the meeting of the American Dental Association; and in case the delegates appointed fail to go, to endeavor to find persons to fill the vacancy.

Dr. Watt moved that two delegates be appointed to convey the greetings of this State Society to the Pennsylvania Society, at their meeting in Pittsburg in April next.

The motion was adopted, and Drs. Geo. Watt and J. Taft appointed by the Chair as said Committee.

On motion of Dr. J. Taft, fifty per cent. of the outstanding accounts against the Society were ordered to be paid out of the funds of the Society now on hand.

On motion of Dr. J. Taft, it was ordered that the remainder of the accounts against the Society, and of the expenses that may occur between this time and the next annual meeting, be paid by the Board of Examiners from any money in their hands.

On motion, the Society then adjourned to meet at Columbus, O., on the first Tuesday of December, 1870.

